# Supplementary material for: Analysis of Pollution of Phthalates in Pork and Chicken in Taiwan Using Liquid Chromatography–Tandem Mass Spectrometry and Assessment of Health Risk
Source: Molecules. 2019 Oct 23;24(21):3817. doi: 10.3390/molecules24213817 (PMC6865204; doi:10.3390/molecules24213817)
Supplement: Supplementary file 1 [file molecules-24-03817-s001.pdf]

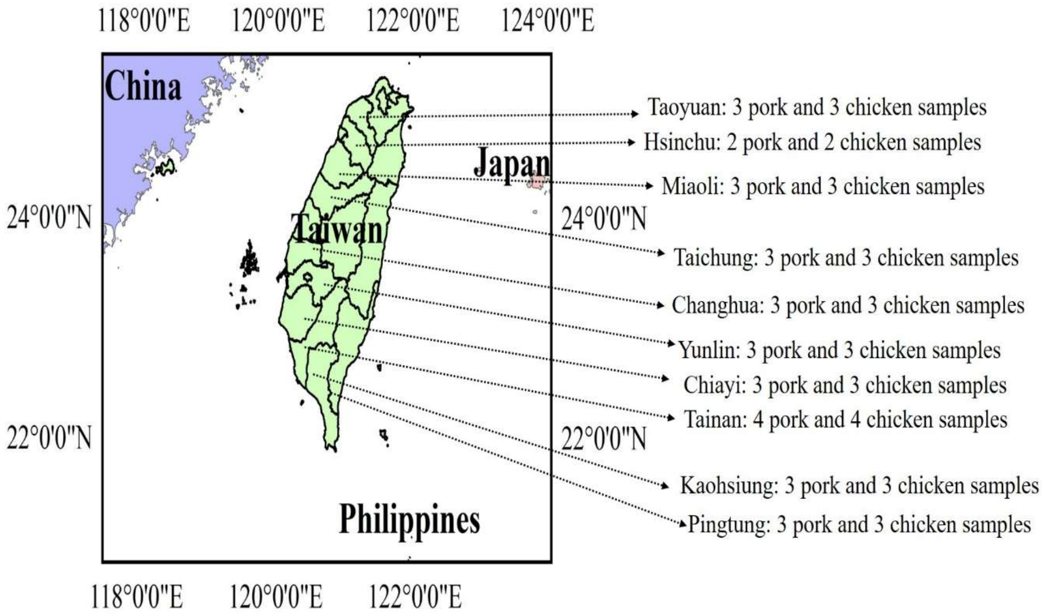

Figure 1. Sampling locations in Taiwan. Sixty samples (30 of pork and 30 of chicken) were collected from major production areas in Taiwan (including Taoyuan, Hsinchu, Miaoli, Yunlin, Taichung, Tainan, Changhua, Chiayi, Hualien, and Kaohsiung).

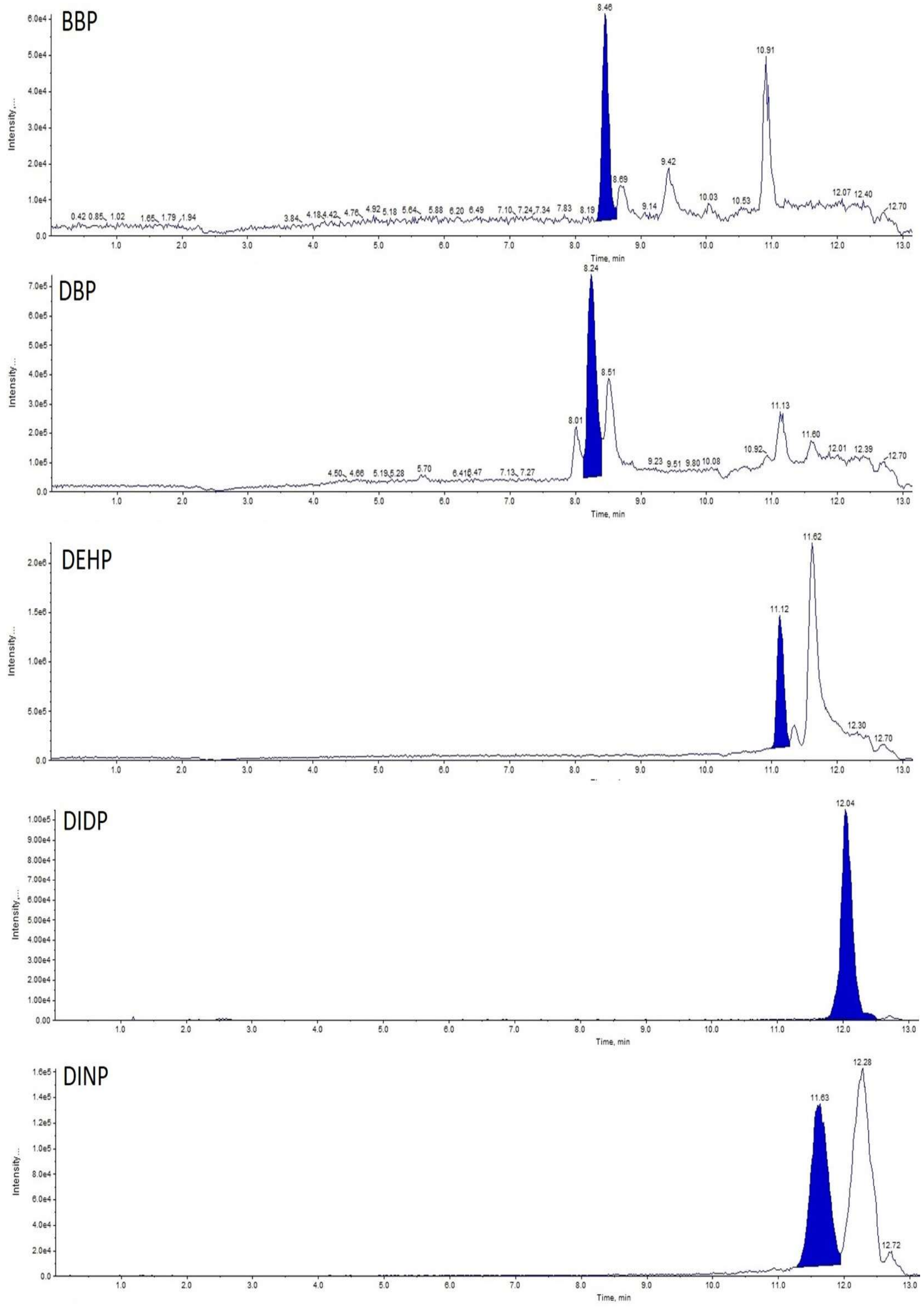

Figure 2. Chromatograms of LC/MS/MS m/z 149 transition products of BBP, DBP, DEHP, DIDP, and DINP in pork matrixes at a limit of detection of 40 ng/g.

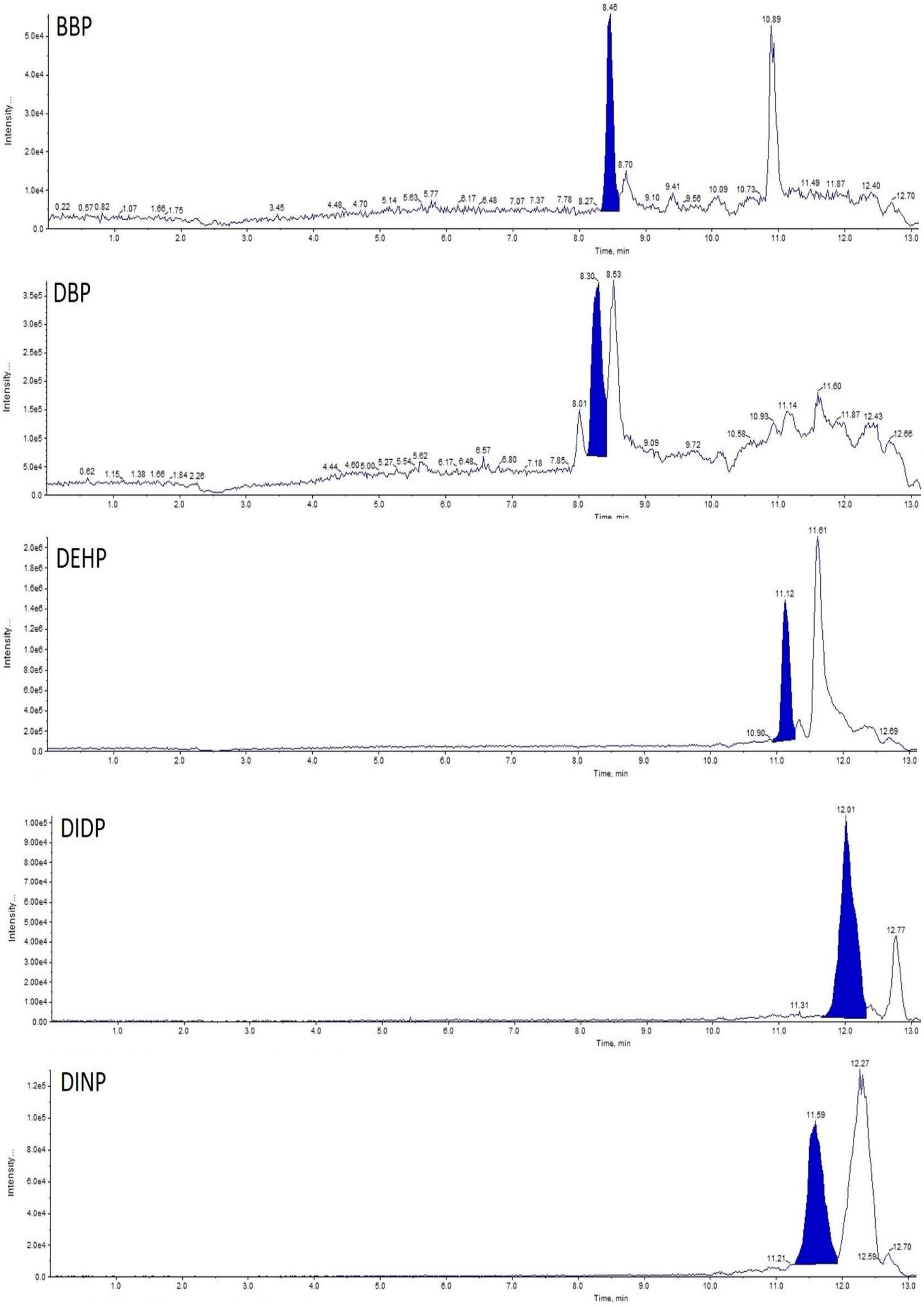

Figure 3. Chromatograms of LC/MS/MS m/z 149 transition products of BBP, DBP, DEHP, DIDP, and DINP in chicken matrixes at a limit of detection of 40 ng/g.

Table 1. Detection ratios of phthalates in pork samples (n = 30) collected during 2016.

Sample (No.)	Phthalates	Detectable Sample	Collected Location	Detectable Ratio (%)	Detected Residues (mg/kg)
1	-	-	Taoyuan	-	-
2	-	-	Taoyuan	-	-
3	-	-	Taoyuan	-	-
4	-	-	Hsinchu	-	-
5	-	-	Hsinchu	-	-
6	-	-	Miaoli	-	-
7	-	-	Miaoli	-	-
8	-	-	Miaoli	-	-
9	-	-	Taichung	-	-
10	-	-	Taichung	-	-
11	-	-	Taichung	-	-
12	-	-	Changhua	-	-
13	-	-	Changhua	-	-
14	-	-	Changhua	-	-
15	-	-	Yunlin	-	-
16	-	-	Yunlin	-	-
17	-	-	Yunlin	-	-
18	-	-	Chiayi	-	-
19	-	-	Chiayi	-	-
20	-	-	Chiayi	-	-
21	DEHP	1	Tainan	3.33	0.80
22	-	-	Tainan	-	-
23	-	-	Tainan	-	-
24<br>25	-	-	Tainan	-	-
25	DEHP	1	Kaohsiung	3.33	0.62
26	-	-	Kaohsiung	-	-
27	-	-	Kaohsiung	-	-
28	-	-	Pingtung	-	-
29	-	-	Pingtung	-	-
30	-	-	Pingtung	-	-
Total		2		6.67	0.05 (Mean)

– Undetectable phthalate compounds, including BBP, DBP, DEHP, DIDP, and DINP.

Table 2. Detection ratios of phthalates in chicken samples (n = 30) collected during 2016.

Sample (No.)	Phthalates	Detectable Sample	Collected Location	Detectable Ratio (%)	Detected Residues (mg/kg)
1	-	-	Taoyuan	-	-
2	-	-	Taoyuan	-	-
3	-	-	Taoyuan	-	-
4	DEHP	1	Hsinchu	3.33	0.43
5	-	-	Hsinchu	-	-
6	-	-	Miaoli	-	-
7	DHEP	1	Miaoli	3.33	0.42
8	-	-	Miaoli	-	-
9	-	-	Taichung	-	-
10	-	-	Taichung	-	-
11	-	-	Taichung	-	-
12	-	-	Changhua	-	-
13	-	-	Changhua	-	-
14	-	-	Changhua	-	-
15	-	-	Yunlin	-	-
16	-	-	Yunlin	-	-
17	-	-	Yunlin	-	-
18	-	-	Chiayi	-	-
19	-	-	Chiayi	-	-
20	-	-	Chiayi	-	-
21	DHEP	1	Tainan	3.33	0.45
22	-	-	Tainan	-	-
23	-	-	Tainan	-	-
24<br>25	-	-	Tainan	-	-
25	-	-	Tainan	-	-
26	-	-	Kaohsiung	-	-
27	-	-	Kaohsiung	-	-
28	-	-	Pingtung	-	-
29	-	-	Pingtung	-	-
30	-	-	Pingtung	-	-
Total		3		10	0.04 (Mean)

– Undetectable phthalate compounds, including BBP, DBP, DEHP, DIDP, and DINP.
